# The impact of a single surfing paddling cycle on fatigue and energy cost

**DOI:** 10.1038/s41598-021-83900-y

**Published:** 2021-02-25

**Authors:** Márcio Borgonovo-Santos, Rodrigo Zacca, Ricardo J. Fernandes, João Paulo Vilas-Boas

**Affiliations:** 1 Centre of Research, Education, Innovation and Intervention in Sport (CIFI2D), Faculty of Sport, University of Porto, Porto, Portugal; 2 Porto Biomechanics Laboratory (LABIOMEP-UP), University of Porto, Porto, Portugal; 3 CAPES Foundation, Ministry of Education of Brazil, Brasília, Brazil; 4 Surfing Viana High Performance Centre – Surf Club Viana, Viana do Castelo, Portugal

**Keywords:** Bioenergetics, Biophysics

## Abstract

Surfing is one additional sport proposed by the Tokyo 2020 Organizing Committee. Surprisingly, substantial efforts to understand surfing energetics are recent, and the impact of a single surfing paddling cycle on fatigue and energy cost is still not clear. Since surfing paddling technique is highly specific, experiments in real practice conditions are necessary to provide deeper insights. Through a biophysical approach, biomechanical and energetics responses of surfing paddling were quantified and compared from 16 competitive male surfers (23.5 ± 10.0 years old, 65.3 ± 11.4 kg and 1.72 ± 0.01 m) during two sets (PRE and POST) of 10 s all-out tethered paddling plus 20 m sprint paddling, interposed by 6 min of endurance paddling. Faster surfers presented lower energy cost during sprint PRE (r^2^ = 0.30, *p* = 0.03) and endurance (r^2^ = 0.35, *p* = 0.02) relative surfing paddling velocities. Although the energy cost was higher for a lower velocity at maximal paddling velocity POST, the energy cost of surfing paddling increased with absolute velocity according to a power function (R^2^ = 0.83). Our results suggest that fatigue seems to occur even following a single surfing paddling cycle. Developing a powerful and endurable metabolic base while reducing energy cost during surfing paddling should be seen as key factors in surfing training programs.

## Introduction

Since the unanimous approval of the International Olympic Committee in 2016, surfing is one additional sport proposed by the Tokyo 2020 Organizing Committee (www.olympic.org/the-ioc). For the first time 20 female and 20 male surfers will compete in Chiba, Japan, as part of the Olympic competition. Seen as a youthful and vibrant sport, surfing is characterized by intermittent bouts of *v*arying durations and intensities, followed by considerable recovery periods^1^. The Hawaiian Duke Kahanamoku, a three-time gold medalist in swimming, widely considered the father of modern surfing, first argued for the sport to be included in the Olympic program in the beginning of the twentieth century (www.tokyo2020.org). Currently, there are millions of active surfers worldwide^1^. Typically, the duration of a surf session ranges from 20 min to 4–5 h in competition and training conditions, respectively^1,2^, with surfers performing, almost cyclically, endurance and sprint paddling, popping-up and maneuvering on the face of the wave^3–5^.

**Figure 1:**
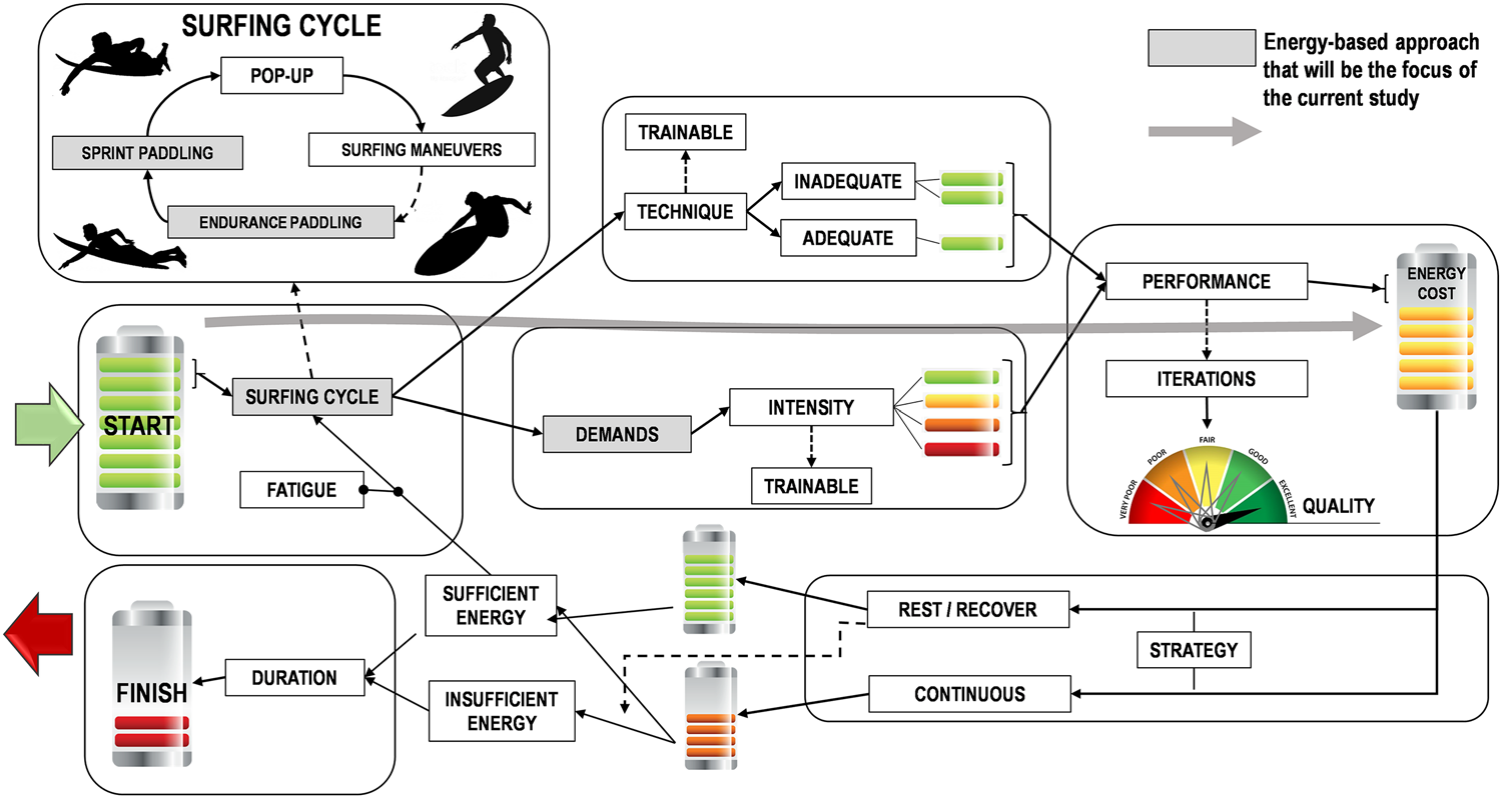
Conceptual model of a surf session energetic profile. Solid grey line indicates an energy-based approach that will be the focus of the current study.

Energetics and technique are relevant performance related factors in surfing^3–5^. Figure 1 illustrates a conceptual model of the main activities during a typical surf session and corresponding energetics. Be exercising or resting during the surf session is a surfer decision to manage fatigue, technique and performance^6–8^.

It is well reported that fatigue impairs muscle performance^7,8^. Thus, depending on the surfing paddling cycle (sprint paddling interposed by endurance paddling) frequency, muscle metabolic homeostasis can be impaired^1,9,10^. During a surf session, surfers expend ~ 50% paddling, ~ 3% wave riding, ~ 7% with miscellaneous (e.g. recovering the surfboard) and ~ 40% recovering. This proportion of tasks is relatively consistent during competition^11–13^, training^9^ or even in recreational practice^14^. The surfing paddling can be divided in paddling to return to the line-up, sprint paddling to the wave and general paddling, with a mean duration of ~ 64, 7 and 15 s and a percentage of ~ 21, 4 and 18%, respectively^9^.

Substantial efforts to better understand surfing energetics are recent^4,10,15^, with the sprint and endurance paddling integrated assessment (combining physiology and biomechanics) being very scarce. This gap is even more evident regarding ecologic-related studies, i.e., conducted in-water^16^. Since surfing paddling technique is highly specific, out-of-water simulations using land ergometers seems to be far from the real effort^17^, reason why experiments in real practice conditions would provide deeper insights about surfers’ energetic profile. Likewise, a biophysical assessment in surfing could better guide researchers and coaches to improve planning strategies and training methods.

The energetics of swimming has been extensively studied^18–20^, providing relevant insights to be applied in surfing related studies^4^. In fact, the velocity in swimming is given by the ratio between net metabolic power (${\dot{\text{E}}}$) and the energy cost to cover a distance unit, with the Aerobic (Aer), anaerobic lactic (AnL) and anaerobic alactic (AnAL) energy contributions depending on the exercise duration and intensity^5,18^. Therefore, in surfing paddling, as in swimming, any influence on hydrodynamic resistance and/or propelling efficiency will lead to changes on energy cost^5,18,20,21^. The current study aimed to understand the impact of a single surfing paddling cycle on fatigue and energy cost. We hypothesized that there seems to be fatigue even in the first surfing paddling cycle.

## Results

**Figure 2:**
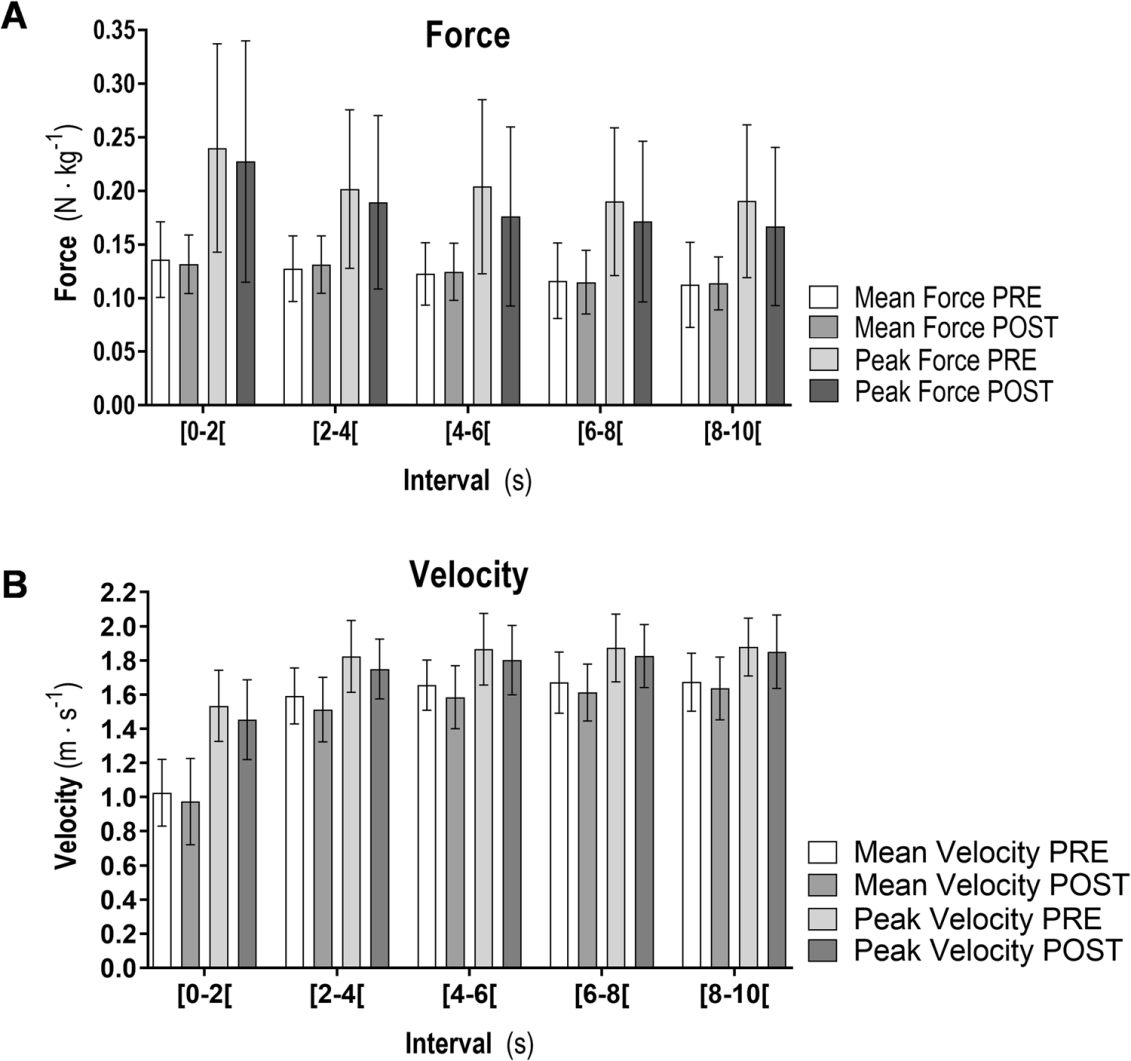
Tethered-paddling force–time performance (**A**) and sprint paddling velocity (**B**) for PRE and POST endurance-paddling. *Non-ordinal force reduction (*p* < 0.01).

No differences were observed for mean tethered paddling force between PRE and POST (0.12 ± 0.01 vs. 0.12 ± 0.01 N kg^−1^) (Fig. 2A). However, a small decrease was observed for mean POST maximal paddling velocity (1.52 ± 0.28 vs. 1.46 ± 0.28 m s^−1^; mean diff: 0.06; 95% IC: 0.005–0.11; *p* = 0.033; Cohen´s *d*: 0.59) (Fig. 2B). 44% of surfers reached their highest velocity in the time interval of [4–6[s at PRE, while at POST 44% of surfers reached higher values on the interval of [6–8[s. Direct relationships between mean tethered paddling force (N kg^−1^) and maximal paddling velocity were not significant when checked separately (PRE: r^2^ = 0.19, *p* = 0.08; POST: r^2^ = 0.14, *p* = 0.15).

**Figure 3:**
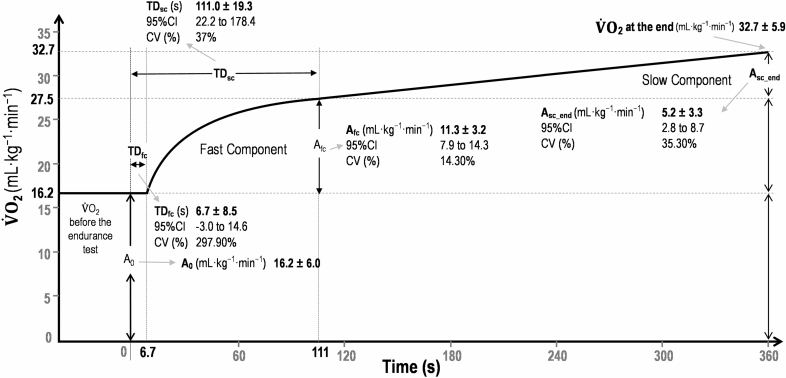
Estimated ${\dot{\text{V}}\text{O}}_{2}$ related parameters (mean ± SD and coefficient of variation) obtained during 6 min paddling at 60% of maximal velocity. A_0_ is the ${\dot{\text{V}}\text{O}}_{2}$ before the endurance test; A_fc_ and A_sc_end_, TD_fc_ and TD_sc_ are respectively amplitudes and corresponding time delays of the fast and slow ${\dot{\text{V}}\text{O}}_{2}$ components. The CV (%) and 95%CI are the mean coefficient of variation and 95% confidence interval for each mean parameter estimate, respectively.

Average distance paddled during 360 s (1.15 ± 0.11 m·s^−1^) was 414 ± 41 m. The HR ranged from 142 ± 23 to 167 ± 17 bpm (74 ± 12 to 88 ± 9% HRmax) in the first and the last minute (respectively). The ${\dot{\text{V}}\text{O}}_{2}$ kinetics response to endurance paddling was best fitted by a bi-exponential model (*p* < 0.05). Estimated ${\dot{\text{V}}\text{O}}_{2}$ related parameters obtained during 6 min paddling at 60% of maximal velocity can be observed in the Fig. 3.

**Figure 4:**
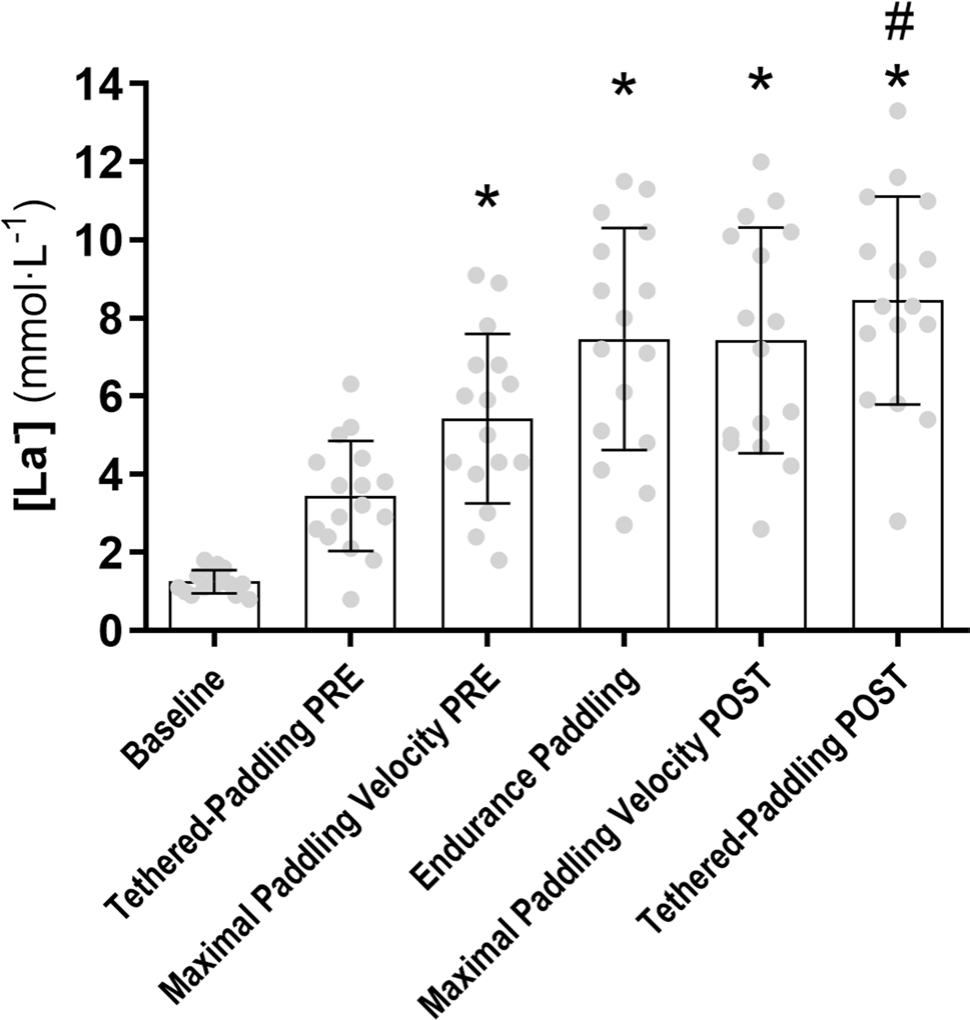
[La^−^] kinetics during the entire protocol. *Difference from tethered-paddling PRE (*p* < 0.05); # Difference from maximal paddling velocity PRE (*p* < 0.05).

The [La^−^] kinetics during the entire protocol and post-hoc analysis from tethered paddling PRE and from maximal paddling velocity PRE are presented in Fig. 4. The Table 1 shows the E_tot_, ${\dot{\text{E}}}$ and energy cost throughout the protocol. Regarding the small decrease in POST maximal paddling velocity, moderate increase were observed for E_tot_ (mean diff: − 8.1 kJ; 95% IC: − 13.4 to 2.8; *p* = 0.005; Cohen´s *d*: − 0.81), ${\dot{\text{E}}}$ (mean diff: − 0.51 kW; 95% IC: − 0.86 to − 0.16; *p* = 0.008; Cohen´s *d*: − 0.77) and energy cost (mean diff: − 0.40 kJ m^-1^; 95% IC: − 0.67 to − 0.14; *p* = 0.005; Cohen´s *d*: − 0.81).

**Table 1:** Energy expenditure throughout the protocol, simulating one surfing cycle.

	Tethered paddling PRE (10 s)	Maximum paddling velocity PRE (20 m)	Endurance paddling (360 s)	Maximum paddling velocity POST (20 m)	Tethered paddling POST (10 s)
Aer energy (kJ)	–	–	247.6 ± 52.7	–	–
AnL energy (kJ)	8.8 ± 6.4	16.3 ± 9.9	25.5 ± 13.5	24.0 ± 14.0	27.5 ± 12.4
AnAL energy (kJ)	9.5 ± 1.7	11.8 ± 2.6	27.4 ± 4.8	12.2 ± 2.7	9.5 ± 1.7
${\text{E}}_{{{\text{tot}}}}$ (kJ)	18.3 ± 7.9	28.1 ± 12.2	300.5 ± 60.5	36.2 ± 16.2	36.9 ± 13.8
${\dot{\text{E}}}$ (kW)	–	2.09 ± 0.79	0.83 ± 0.16	2.60 ± 1.06	–
*C* (kJ m^−1^)	–	1.40 ± 0.61	0.73 ± 0.17	1.81 ± 0.81	–

*Aer* aerobic, *AnL* anaerobic lactic, *AnAL* anaerobic alactic, *E*_*tot*_ total energy expenditure, $\dot{E}$: metabolic power, *C* energy cost, *-* not estimated.

Direct relationships between body mass and energy cost were observed at sprint (PRE: r^2^ = 0.70, *p* < 0.001; POST: r^2^ = 0.71, *p* < 0.001) and endurance paddling (r^2^ = 0.40, *p* < 0.008). However, the direct relationship between body mass and velocity was not significant in any test.

**Figure 5:**
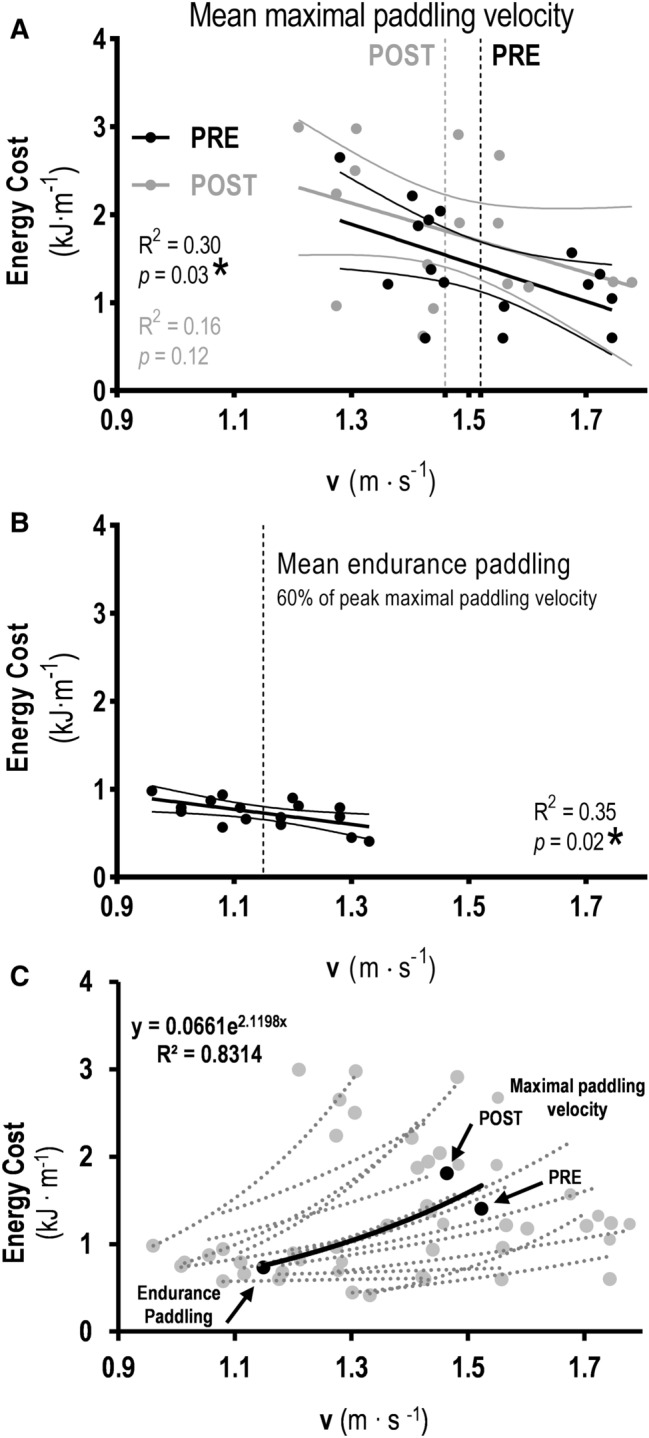
Energy cost at two different relative intensities, maximal paddling velocity (PRE and POST; **A**) and endurance paddling (**B**), and energy cost versus absolute velocity relationship (**C**) for all surfers.

Figure 5A,B present the economy profile (energy cost) at two different relative intensities: maximal paddling velocity (PRE and POST) (panel A) and endurance paddling for all surfers (panel B). The energy cost decreased with maximal paddling velocity PRE (r^2^ = 0.30, *p* = 0.03) and endurance paddling (r^2^ = 0.35, *p* = 0.02), but not in maximal paddling velocity POST (r^2^ = 0.16, *p* = 0.12). Thus, faster surfers presented lower energy cost during sprint (PRE) and endurance velocities. Figure 5 panel C, presents the energy cost versus absolute velocity relationship for the surfers from the present study. Although the energy cost was higher for a lower velocity at maximal paddling velocity POST, the energy cost of surfing paddling increased with velocity according to a power function, being described by the following equation:

1 $${\text{y}} = {0}{\text{.0661e}}^{{{2}{\text{.1198x}}}} {;}\,{\text{R}}^{{2}} = {0}{\text{.8314}}$$

## Discussion

We performed a surfing biophysical analysis to understand the biomechanics and energetics of sprint and endurance paddling during a single surfing paddling cycle. We characterized the biomechanics and energetics of a surfing paddling cycle, comparing sprint paddling performances interposed by endurance paddling in competitive male surfers. The protocols were conducted respecting an ecological approach for surfing paddling, simulating real efforts in-water. Small impairment was observed for mean maximal paddling velocity POST, despite similar values for mean tethered paddling force. Relationships between mean tethered paddling force (N kg^−1^) and maximal paddling velocity were not significant. The ${\dot{\text{V}}\text{O}}_{2}$ kinetics in response to endurance paddling was best fitted by a bi-exponential model and mean [La^−^] increased during the surfing paddling cycle. Moderate increase were observed for E_tot_, ${\dot{\text{E}}}$ and energy cost. Faster surfers presented lower energy cost during sprint (PRE) and endurance relative velocities. Although the energy cost was higher for a lower velocity at maximal paddling velocity POST, the energy cost of surfing paddling increased with velocity according to a power function. It was evident that there seems to be accumulated fatigue^6–8^ even in a single typical surfing paddling cycle, supporting our hypothesis and improving the knowledge on biomechanics and energetics during a surf session.

Tethered swimming is considered a valid procedure for swimmer’s propulsive force assessment and reliable to estimate swimming performance^22^. However, the relationships between mean tethered paddling force (N kg^−1^) and maximal paddling velocity, checked separately in our study (both in PRE and POST moments), were not significant. In fact, although tethered paddling does not evaluate the hydrodynamic drag that the surfer and surfboard system must overcome, it allows assessing the surfer's capacity to produce force. A similar protocol in swimming (10 s maximal tethered bouts) found a peak force of 207.1 ± 27.2 N and mean force of 133.2 ± 16.8 N^23^. Smaller absolute force values (Fig. 2, panel A) were observed (peak force of 163.9.1 ± 44.5 N and mean force of 76.7 ± 18.7 N). These differences were expected, firstly due to surfers’ age and anthropometrics heterogeneity. Secondly, the surfboard buoyancy makes only part of the arms to be immersed in the water during the propulsion phase, thus reducing the water contact area, a mechanical determinant to produce propulsion force in water locomotion^24^.

The effect of wind, water surface and currents, which affect the surfers’ performance, can be controlled in a swimming pool environment^25^. Besides, surfing evaluation protocols in swimming pool environments are more respectful (ecological) when comparing the characteristics of movements performed by surfers into the ocean with land ergometers^26^. Pool-based protocols have already been applied in junior practitioners and professional surfers (peak velocities ranging from 1.10 m s^−1^ to 2.00 m s^−1^)^9,10,15,27–30^. Our results are in agreement with the available literature (Fig. 2, panel B). However, we also tested the effect of endurance paddling (and recovery intervals) on the performance of sprint paddling (POST). Impaired mean and peak velocity suggest that repeated cycles may drop sprint paddling performance. This information may help to improve coaches’ strategies and training programs.

To date, evaluating the surfing endurance paddling energetic profile (and also sprint paddling) in real environment (sea) and with direct measurements was not possible. Besides, the portability of available equipment limits the area of data collection, thus creating some movement constraints for surfers. This is why studies are typically conducted with tethered surfboard paddling^31^, adapted ergometers on land^12,13,15,29,30,32^ and, recently, using a swim flume^16^. However, tests performed on land ergometers may underestimate efforts from water locomotion^33^.

Studies evaluating surf sessions in ecological conditions are scarce. Mean velocities of ~ 52 m∙min^−1^ were reported by Secomb et al.^9^, resulting in ~ 312 m during six minutes (counting recovery time dilution). The endurance-paddling test in our study was fixed in six minutes effort, with quite higher distances (414 ± 41 m; 1.15 ± 0.11 m s^−1^). In our study, surfers reached ~ 74% HR_max_ after the first minute effort and ~ 88% HR_max_ after 6 min effort, with mean and maximal HR values being comparable with other studies^9,14^.

This is first study detailing the ${\dot{\text{V}}\text{O}}_{2}$ kinetics in surfing paddling. The lower velocity observed at POST indicates that fatigue can occur even during a single surfing paddling cycle. Indeed, a bi-exponential model best modelled the ${\dot{\text{V}}\text{O}}_{2}$ kinetics in response to endurance paddling, i.e., a loss of muscle metabolic homeostasis may have been observed, impairing muscle power production. Thus, the recruitment of extra motor units leads to a higher energy cost, which will develop a slow component in the ${\dot{\text{V}}\text{O}}_{2}$ kinetics^6–8^. Endurance paddling was performed at 60% of the peak velocity previously obtained, with lower values than those from Furness et al.^15^ who analyzed competitive and recreational surfers. Other studies^27,28^ showed ~ 37 ml kg^−1^·min^−1^ as the lower ${\dot{\text{V}}\text{O}}_{2}$ peak value during maximal surfing paddling.

Maximal surfing paddling velocity is as greater the higher the ${\dot{\text{E}}}$ and the lower the energy cost of the surfer^5,21^. The ${\dot{\text{E}}}$ results from energy sources, and energy cost relies on hydrodynamic resistance, overall efficiency and propelling efficiency^5,21^. Thus, factors influencing energy cost (e.g. velocity, paddling rate/length, body mass, training) affects drag, propelling efficiency or both^5,18,21,34^. In the present study, maximal paddling velocity POST was lower than PRE, but ${\dot{\text{E}}}$ and energy cost increased. The relationships between energy cost and velocities close to those obtained during actual competitions have been studied for swimming and boat locomotion^5,21^, opening the window to explore it, for the first time, in surfing paddling.

Both in swimming and boat locomotion, there is a direct relationship between energy cost and hydrodynamic resistance, and an inverse relationship between propelling efficiency and overall efficiency^5,21^. In our study the energy cost was analyzed at both relative and absolute velocities. Regarding relative velocities, lower energy cost was associated with higher velocities in male surfers at both maximal paddling velocity and endurance paddling (Fig. 5 panel A and B). Although energy cost was higher for a lower absolute velocity at maximal paddling velocity POST (Fig. 5 panel C), the energy cost of surfing paddling increased with velocity according to a power function^5,21,35^. The relationship between body mass and velocity was not significant, but the direct relationships between body mass and energy cost were strong. Thus, the lower energy cost observed for faster surfers is, at least in part, due to the smaller hydrodynamic resistance (form, friction and wave drag), i.e., a lower wetted area of the surfer and his board and a more horizontal position in water^5,21^. Besides, the distance covered per stroke cycle (in swimming) or paddling cycle (in surfing and boats) is related to the efficiency of locomotion, i.e. the higher the distance covered, the higher the propelling and overall efficiencies^35^. In fact, energy cost in boats is lower when comparing to swimming, due to a lower hydrodynamic resistance and higher propelling efficiency^5^. The increase in ${\dot{\text{E}}}$ with velocity is associated with the increase in total mechanical power output from muscles to sustain that velocity, which means that powerful and endurable metabolic base cannot be overlooked in surfing paddling training programs^36^. Despite that, improvements in velocity can easily be reached by reducing energy cost rather than by increasing ${\dot{\text{E}}}$ (from aerobic or anaerobic pathways) by the same amount^5,21,37^. For that purpose, technique training and hydrodynamics of the surfboard are mandatory issues.

The sequence of experiments from our study was created to simulate, in part (sprint and endurance paddling), the real conditions of a surf session. Differences in [La^−^] were observed between surfing paddling sprint and endurance paddling. Together, the results from our study confirm the initial hypothesis, i.e. fatigue can occur after the first cycle of sprint and endurance paddling, which could affect subsequent surfing cycles, even with the available rest intervals. Our work examined surfers’ performance in a swimming pool, performing the paddling action at different intensities, which is somewhat different from paddling in the ocean. For instance, salt water generates greater buoyancy forces relative to fresh water, and these differences may contribute to differences in the total energy expenditure. In addition, the tethered force test (stationary paddling) allowed us to measure the upper limbs paddling propulsion. However, limitations in equipment have not allowed us to test it in the ocean. On the other hand, the analyses from our study were performed under highly controlled and repeatable conditions, which are very difficult to achieve in the ocean. Besides, the paddling motions analyzed in this study are biomechanically and energetically very similar, apparently, to those performed in open water.

In conclusion, our findings suggest that there seems to be fatigue even in a single typical surfing paddling cycle. Although faster surfers seem to be more economical at both maximal paddling velocity and endurance paddling, the energy cost of surfing paddling increased with velocity according to a power function. Together, these findings offer an original assessment of bioenergetics during sprint and endurance paddling, respecting the ecology of surfing. The endurance test between sprint paddling, simulating a surfing paddling cycle, brought new insights into the ${\dot{\text{V}}\text{O}}_{2}$ kinetics of surfers during the paddling action, best fitted by a bi-exponential behavior with fast and slow components. Finally, the entire protocol showed an estimation of the energy pathways, E_tot_, ${\dot{\text{E}}}$ and energy cost during a single surf paddling cycle, contributing to better understand the biomechanical and energetic requirements in surfing. The chance of fatigue installation already in the first surfing paddling cycle provide valuable knowledge, which can be applied in surfing for training, testing and competitions. From a strategy point of view, coaches can estimate E_tot_, ${\dot{\text{E}}}$ and energy cost to better allocate recoveries, surfing paddling intensities and technique, whether in a recreation, training or competition.

## Methods

**Figure 6:**
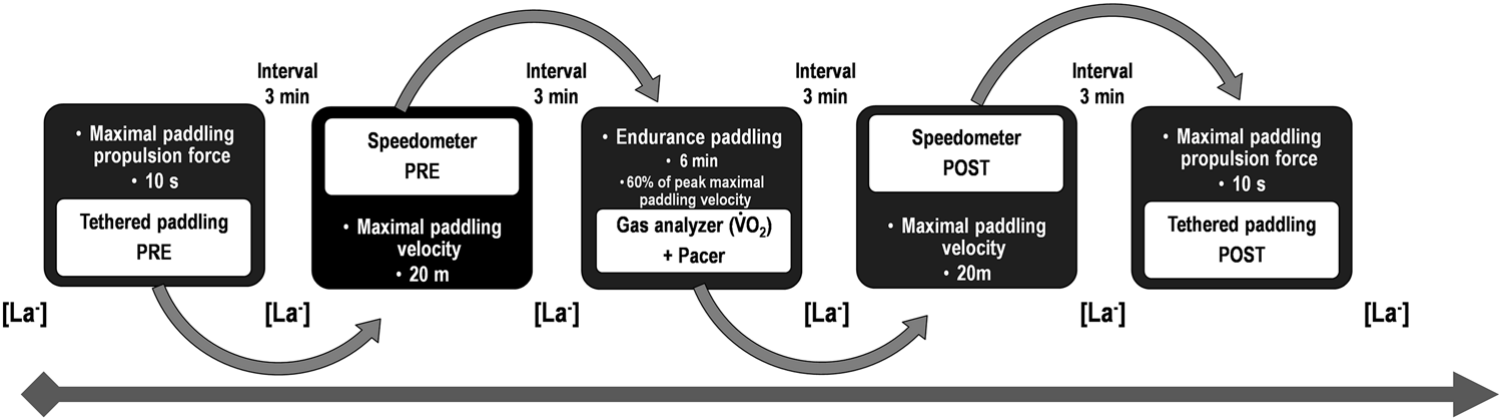
Design of the study in which surfers were tested trough specific functional protocols of sprint (PRE and POST) and endurance paddling actions, thus simulating a surfing paddling cycle. [La^−^]: blood lactate sample assessment.

A single-group prospective study was conducted, in which surfers were tested trough specific functional protocols, simulating the combination of sprint and endurance paddling actions during one surfing cycle. The experimental protocol took place in a 25 m indoor pool with 27 and 26ºC of water and air temperatures (respectively) and 65% relative humidity. Surfers used their own surfboards, minimizing eventual constraints and simulating their personal surfing environment. After a warm-up (with self-stretching exercises, 3 min moderate intensity continuous paddling, 2 × 15 m maximal intensity paddling and 10 min of recovery)^30^, surfers performed a sequence of functional paddling tests (Fig. 6) consisted by sprint (PRE and POST) and endurance paddling protocols (with 3 min of rest intervals in-between tests). The proposed warm-up, typically carried out in experiments with surfers^30^, tries to maintain the ecology of what normally takes place prior to a surf session, since most of the warm-up is performed out of the water.

### Participants

Sixteen competitive male surfers (age: 23.5 ± 10.0 years, body mass: 65.3 ± 11.4 kg, height: 1.72 ± 0.01 m, arm span: 1.75 ± 11.4 m and 9.1 ± 8.9 years of previous experience) volunteered to participate. The inclusion criteria were two years of surfing experience, two training sessions per week of regular practice and the absence of any serious musculoskeletal injury in the last six months. Surfers and respective parents (when subjects were under 18 years old) were informed about the benefits and risks of taking part in experiments. After receiving all information about the data collection protocols so that they could participate in the study, all participants gave their duly signed informed consent forms. This study was approved by the ethics committee of the Faculty of Sport, University of Porto—Porto—Portugal (CEFADE 27.2014) and the procedures were carried out in accordance of Declaration of Helsinki.

### Procedures

**Figure 7:**
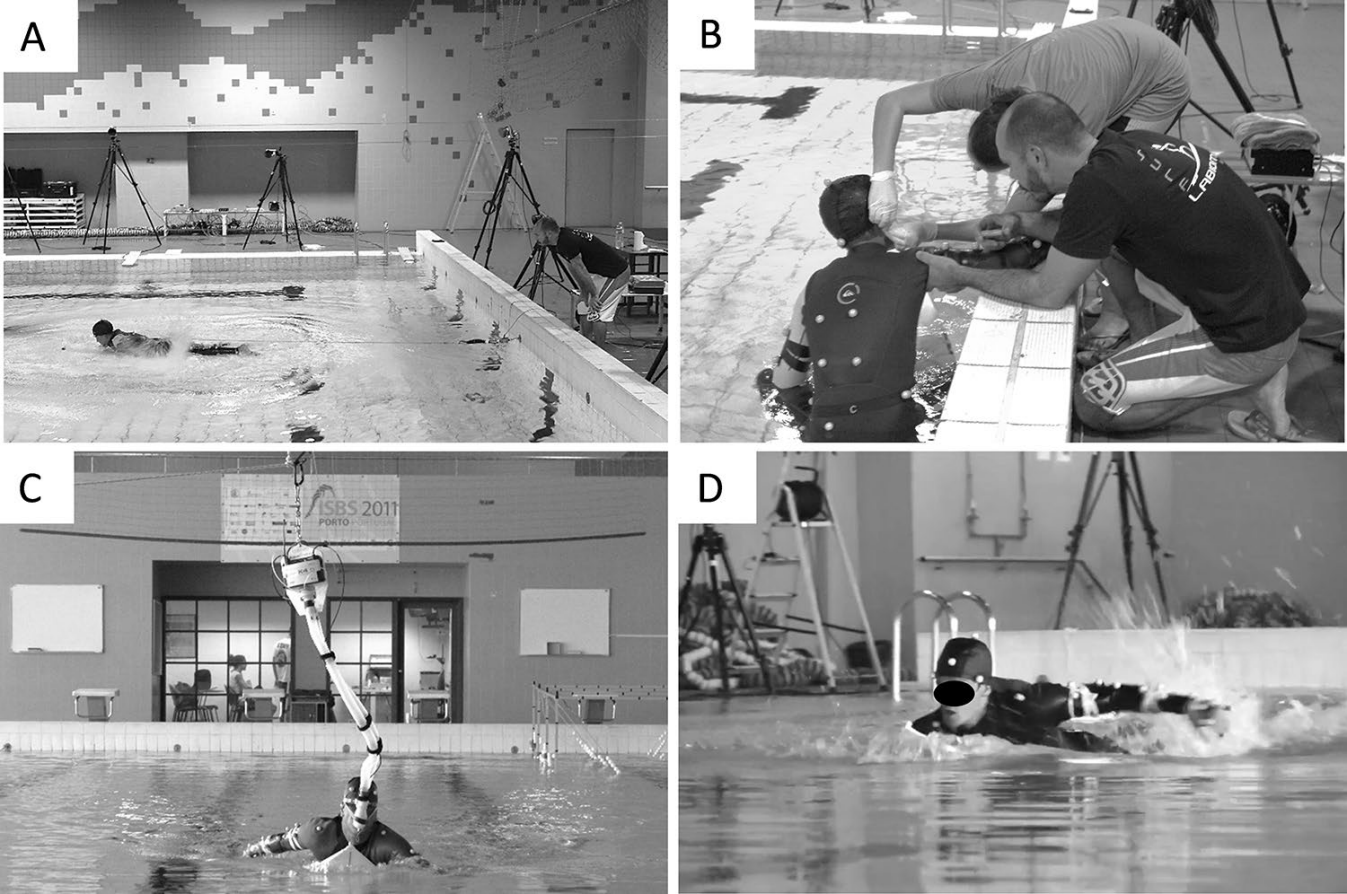
Tests on the swimming pool: (**A**) tethered paddling; (**B**) blood lactate assessment; (**C**) endurance paddling test with ${\dot{\text{V}}\text{O}}_{2}$ assessment and heart rate monitoring; (**D**) maximal paddling velocity test.

The anthropometric profile (body mass, height and arm span) was obtained by an International Society for the Advancement of Kinanthropometry accredited level I anthropometrist. For assessing the maximal paddling propulsive force, a belt (attached on the lumbar-sacral area) was connected to a 5 m length non-elastic steel cable. The cable was coupled to a load cell (5000 N, Globus, Codogne, Italy; 100 Hz frequency) fixed on the wall^22^ connected to an analogic/digital data acquisition system (Biopac MP150 and software AcqKnowledge 4 (BIOPAC Systems, Inc., Goleta, CA, USA). A digital low-pass filter smoothed data with 10 Hz cut-off frequency to remove noise and movement artefacts was used. Surfers adopted a horizontal position on the surfboard with the cable fully extended (Fig. 7, panel A), with data collection starting when the first paddle cycle was completed, avoiding the cable extension inertial effect^22^. Since the sprint paddling duration is ~ 7 s^9^, each participant performed the all-out tethered surfing paddling test during 10 s^29,30^.

Then, to assess maximal paddling velocity, subjects performed a 20 m all-out paddling test, starting in a prone position on the surfboard without contact with the wall (Fig. 7, panel D). Velocity was recorded using a custom-made cable-based speedometer device fixed at the wall, 0.3 m above the water surface^38^, using a bobbin with a non-elastic line fixed at the lumbar region. Continuous velocity data was obtained at a 50 Hz frequency, exported to the software AcqKnowledge 4 (BIOPAC Systems, Inc., Goleta, CA, USA) and filtered with a 15 Hz cut-off digital filter (FIR—Window Blackman − 61 dB). The cut-off value was selected using Fast Fourier Transform analysis to minimize artefact noise, with peak and mean velocities obtained by individual velocity–time curves every two seconds. The second trial (POST) of both the above-referred tests was performed after the endurance paddling test using the same methodological procedures.

Paddling bouts can last from few to ~ 90 s during a 2 h training session^9^ and the endurance paddling mean velocity ranges from 64 to 70% of maximal velocity^27,28,39^. Thus, each surfer paddled 6 min in the endurance paddling test, at 60% of the peak velocity previously obtained. For that purpose, two cones were placed on the edge of the pool (20 m away from each other) as a reference for surfers inverting their direction and to guarantee they performed at constant velocity (using acoustic pacing).

Respiratory and pulmonary gas exchange data were measured breath-by-breath using a telemetric portable gas analyzer (K4b^2^, Cosmed, Rome, Italy) connected to a respiratory snorkel and valve system (AquaTrainer, Cosmed, Rome, Italy)^40^—Fig. 7, panel C. The K4b^2^ was calibrated prior to each testing session with gases of known concentration (16% O_2_ and 5% CO_2_) and the turbine volume transducer was calibrated using a 3 L syringe according to the manufacturer instructions. The telemetric portable gas analyzer was transported along the swimming pool suspended at a 2 m height over the water on a steel cable^41^. The ${\dot{\text{V}}\text{O}}_{2}$ kinetics parameters were estimated, including the precision of estimation (confidence limits), by bootstrapping with 1000 samples^42,43^. The cardiodynamic phase was not considered for ${\dot{\text{V}}\text{O}}_{2}$ kinetics analysis. Parameter estimates and the goodness of fit of each model (mono- and bi-exponential) were only analyzed with raw data^43^. For each surfer, the on-transient of endurance paddling test was modelled with mono- and bi-exponential models using the VO_2_FITTING software^43^, as described in Eqs. (2) and (3):

2 $${\dot{\text{V}}\text{O}}_{2} \left( {\text{t}} \right) = {\text{A}}_{0} + {\text{H}}\left( {{\text{t}} - {\text{TD}}_{{{\text{fc}}}} } \right) \times {\text{A}}_{{{\text{fc}}}} \left( {1 - {\text{e}}^{{ - ({\text{t}} - {\text{TD}}_{{{\text{fc}}}} )/\uptau _{{{\text{fc}}}} }} } \right)$$

3 $${\dot{\text{V}}\text{O}}_{2} \left( {\text{t}} \right) = {\text{A}}_{0} + {\text{H}}\left( {{\text{t}} - {\text{TD}}_{{{\text{fc}}}} } \right) \times {\text{A}}_{{{\text{fc}}}} \left( {1 - {\text{e}}^{{ - ({\text{t}} - {\text{TD}}_{{{\text{fc}}}} )/\uptau _{{{\text{fc}}}} }} } \right) + {\text{H}}\left( {{\text{t}} - {\text{TD}}_{{{\text{sc}}}} } \right) \times {\text{A}}_{{{\text{sc}}}} \left( {1 - {\text{e}}^{{ - ({\text{t}} - {\text{TD}}_{{{\text{sc}}}} )/\uptau _{{{\text{sc}}}} }} } \right)$$

where ${\dot{\text{V}}\text{O}}_{2}$(t) represents the ${\dot{\text{V}}\text{O}}_{2}$ normalized to body mass at the time t, A_0_ is the ${\dot{\text{V}}\text{O}}_{2}$ at rest (2 min average) and H represents the Heaviside step function described in Eq. (4)^44^. The A_fc_ and A_sc_, TD_fc_ and TD_sc_, and τ_fc_ and τ_sc_, are the amplitudes, the corresponding time delays and time constants of the fast and slow ${\dot{\text{V}}\text{O}}_{2}$ components, respectively.

4 $${\text{H}}\left( {\text{t}} \right) = \left\{ {\begin{array}{*{20}c} {0,} & {t < 0} \\ {1,} & {t \ge 0} \\ \end{array} } \right.$$

${\dot{\text{V}}\text{O}}_{2}$ at the end was calculated as the average of the last 60 s of exercise. Since the asymptotic value of the second function is not necessarily reached at the end of the exercise, the amplitude of the A_sc_ at the end of the test (A_sc_end_) was also calculated (Eq. 5)^45^:

5 $${\text{A}}_{{{\text{sc}}\_{\text{end}}}} = {\text{A}}_{{{\text{sc}}}} (1 - {\text{e}}^{{ - ({\text{t}}_{{{\text{end}}}} - {\text{TD}}_{{{\text{sc}}}} )/\uptau _{{{\text{sc}}}} }} )$$

where t_end_ is the time at the end of the endurance paddling test. Heart rate (HR) was continuously monitored using a Polar Vantage NV (Polar Electro Oy, Kempele, Finland) transmitting data telemetrically to the K4b^2^ portable unit aiming to assess the percentage of maximal HR (HRmax) during the effort. Capillary blood samples for blood lactate concentrations ([La^−^]) assessment using a Lactate Pro analyzer (Arkay, Inc., Kyoto, Japan) were collected from the earlobe (Fig. 7, panel B) before exercise, during the recovery intervals and immediately after the tests (at the first and third min). During sprint and endurance paddling tests surfers received verbal encouragement to be motivated and achieve their best performance.

The total energy expenditure (${\text{E}}_{{{\text{tot}}}}$) during the 6 min endurance paddling was estimated by the sum of anaerobic alactic (AnAL), anaerobic lactic (AnL) and aerobic (Aer) energy pathways^5,18,36^. The ${\text{E}}_{{{\text{tot}}}}$ during sprint paddling was estimated by the sum of AnAL and AnL energy pathways due to its short time duration (~ 10 s)^46^. The Aer energy expenditure was calculated from the time integral of the net ${\dot{\text{V}}\text{O}}_{2}$ versus time relationship (di Prampero 1986; Zamparo et al. 2020) and AnL obtained using the following equation^47,48^:

6 $${\text{AnL}} = \left[ {{\text{La}}^{ - } } \right]_{{{\text{net}} }} \cdot\upbeta \cdot {\text{M}}$$

where [La^−^]_net_ is the difference between the [La^−^] before and after exercise, β is the constant for O_2_ equivalent of [La^−^]_net_ (2.7 ml·kg^−1^·mM^−1^) and M is the body mass^47^. Then, AnL was expressed in kJ by assuming an energy equivalent of 20.9 kJ L^−1^^47^. AnAL was estimated from the maximal phosphocreatine splitting in the contracting muscle using the equation^48^:

7 $${\text{AnAL}} = {\text{PCr}} \cdot \left( {1 - {\text{e}}^{{ - {\text{t}}/\uptau }} } \right) \cdot {\text{M}}$$

where PCr is the phosphocreatine concentration at rest, t is the exercise time, τ is time constant of the PCr splitting at exercise onset (23.4 s) and M is the body mass. Subsequently, it was expressed in kJ by assuming an energy equivalent of 0.468 kJ·mM^−1^ and a phosphate/oxygen ratio of 6.25^49^. The energy cost was obtained as the ratio between ${\text{E}}_{{{\text{tot}}}}$ and distance, and the metabolic power (${\dot{\text{E}}}$) was estimated as the ratio between ${\text{E}}_{{{\text{tot}}}}$ and time^34,36,49^.

### Statistical analyses

An algorithm for identifying maximal and minimum force–time curve peaks was developed in the Excel 2013—VBA package (Microsoft Corp., Redmond, WA). Mean and SD are presented as descriptive statistics. Normality, homogeneity and sphericity were satisfied. Repeated Measures ANOVA was applied to check differences between PRE and POST sprint-paddling tests. When necessary Bonferroni *post-hoc* was used. Effect sizes (Cohen’s *d*) were interpreted with the following criteria: 0–0.19 trivial, 0.2–0.59 small, 0.6–1.19 moderate, 1.2–1.99 large, 2.0–3.99 very large and > 4.0 nearly perfect^50,51^. Linear and non-linear regressions between biomechanical and energetic variables were computed. Statistical analysis was carried out using Statistica 12 software (StatSoft, Tulsa, USA). A significance level (α) of 0.05 was defined a priori.

## Ethical approval

All procedures performed in studies involving human participants were in accordance with the ethical standards of the institutional and/or national research committee and with the 1964 Helsinki declaration and its later amendments or comparable ethical standards.

## Informed consent

Informed consent was obtained from all individual participants included in the study.

## Data Availability

Raw and unprocessed data are available upon request at rzacca@fade.up.pt.
